# Comparing genotyping algorithms for Illumina's Infinium whole-genome SNP BeadChips

**DOI:** 10.1186/1471-2105-12-68

**Published:** 2011-03-08

**Authors:** Matthew E Ritchie, Ruijie Liu, Benilton S Carvalho, The Australia and New Zealand Multiple Sclerosis Genetics Consortium (ANZgene), Rafael A Irizarry

**Affiliations:** 1 Bioinformatics Division, The Walter and Eliza Hall Institute of Medical Research, 1G Royal Parade, Parkville, Victoria 3052, Australia; 2 Department of Medical Biology, The University of Melbourne, Parkville, Victoria 3010, Australia; 3 Department of Oncology, University of Cambridge, CRUK Cambridge Research Institute, Li Ka Shing Centre, Robinson Way, Cambridge CB2 0RE, UK; 4 Department of Biostatistics, Johns Hopkins Bloomberg School of Public Health, North Wolfe Street E3035, Baltimore, MD 21205, USA

## Abstract

**Background:**

Illumina's Infinium SNP BeadChips are extensively used in both small and large-scale genetic studies. A fundamental step in any analysis is the processing of raw allele A and allele B intensities from each SNP into genotype calls (AA, AB, BB). Various algorithms which make use of different statistical models are available for this task. We compare four methods (GenCall, Illuminus, GenoSNP and CRLMM) on data where the true genotypes are known in advance and data from a recently published genome-wide association study.

**Results:**

In general, differences in accuracy are relatively small between the methods evaluated, although CRLMM and GenoSNP were found to consistently outperform GenCall. The performance of Illuminus is heavily dependent on sample size, with lower no call rates and improved accuracy as the number of samples available increases. For X chromosome SNPs, methods with sex-dependent models (Illuminus, CRLMM) perform better than methods which ignore gender information (GenCall, GenoSNP). We observe that CRLMM and GenoSNP are more accurate at calling SNPs with low minor allele frequency than GenCall or Illuminus. The sample quality metrics from each of the four methods were found to have a high level of agreement at flagging samples with unusual signal characteristics.

**Conclusions:**

CRLMM, GenoSNP and GenCall can be applied with confidence in studies of any size, as their performance was shown to be invariant to the number of samples available. Illuminus on the other hand requires a larger number of samples to achieve comparable levels of accuracy and its use in smaller studies (50 or fewer individuals) is not recommended.

## Background

In the past decade, hundreds of studies investigating the genetics of common human diseases have been published [1]. High-density SNP microarrays cataloguing variation identified in the HapMap project [2] have been the enabling technology behind these large-scale genome-wide association studies. These microarrays allow the collection of genotypes for many SNPs in many individuals at relatively low cost. The two major producers of these microarrays are Affymetrix Inc. (Santa Clara, CA) and Illumina Inc. (San Diego, CA). The platforms offered by these companies differ substantially in terms of array fabrication, probe design, sample preparation and hybridization protocol. However, both currently genotype around 1 million SNPs per sample and also include non-polymorphic probes for assessing copy number variation in the genome.

Illumina's BeadChips have rapidly increased in both SNP density (from 100,000 to 1,000,000 SNPs) and in the number of samples processed in parallel (1, 2, 4, 8 or 12 per BeadChip) over the past few years. Illumina whole-genome SNP BeadChips use Infinium chemistry, which differentially labels allele A and allele B with red and green dye respectively [3,4]. A number of algorithms are available for processing the raw signal from these arrays into genotype calls. These methods include: GenCall [5], Illumina's proprietary method implemented in the BeadStudio/GenomeStudio software; Illuminus [6]; GenoSNP [7]; CRLMM [8-10]; Birdseed, available in the Birdsuite software [11]; and BeagleCall [12].

In this paper we compare the four widely applicable methods GenCall, Illuminus, GenoSNP and CRLMM on different data sets, measuring performance in terms of accuracy and the ability of each method to flag poor quality calls, SNPs and samples.

## Methods

### Algorithms

The four genotype calling methods we compare vary in their modelling approaches and assumptions. Table 1 summarizes the major features of each algorithm in terms of normalization method, underlying model and computing platform supported. The main modelling differences lie in the normalization method and clustering. Normalization can occur either within sample (GenCall, Illuminus, GenoSNP), or both within and between samples (CRLMM). Likewise, the model-based clustering can occur within sample (GenoSNP) or between samples (GenCall, Illuminus, CRLMM). In the description below, we use Illumina's nomenclature of X and Y to refer to the intensities for the respective alleles (in general X = allele A and Y = allele B).

**Table 1:** Summary of the genotyping algorithms compared.

Method	Major Features		
	Normalization	Model	System
GenCall	W	B	Win

Illuminus	W	B	Lin

GenoSNP	W	W	Lin/Win

CRLMM	W+B	B	Win/Lin/OSX

**Key: **W - within sample method; B - between sample method; Win - Windows operating system; Lin - Linux operating system; OSX - Macintosh operating system

GenCall is the standard vendor provided method from Illumina [5] which is available as a module in the BeadStudio/GenomeStudio software. After reading in the data from binary files (idats) produced by Illumina's scanning system, normalization using an affne transform to rotate and re-scale the X and Y intensities is applied to decrease dependence between the two alleles [4]. Normalization is performed separately for beads from different 'bead pools'. A 'bead pool' refers to a set of beads that have been manufactured together and are located in roughly the same physical position (strip) on a BeadChip. Polar coordinates (R, *θ*) are calculated from the normalized X and Y values. Clustering is performed by the GenTrain algorithm, which is a between sample model. Neural networks which take the polar coordinate transformed data and estimate the SNP-specific centroids for each genotype are used. Default cluster centroids are calculated using data from a set of HapMap samples [2] (Table 2). Alternatively, users may perform clustering using the available samples to calibrate the cluster positions to the data. Genotypes are then assigned by determining the nearest cluster. The GenCall score (GC) is a confidence measure assigned to each call which can be used to filter poor quality calls, SNPs or samples. Illumina generally recommend that calls with GC ≤ 0.15 represent failed genotypes. Averaged GC scores over all SNPs from a given sample, or across all samples for a given SNP can be used as sample or SNP quality metrics. A more commonly used sample quality metric is the 'no call rate'. For GenCall, genotypes with GC score less than a given threshold (0.15 in our analyses) are declared as missing. The proportion of missing values, or 'no calls' in each sample gives the no call rate; samples with higher rates are deemed less reliable than samples with lower rates. No call rates less than 1% should be expected for good quality samples which have been properly processed (Illumina Technical Support, personal communication).

A second alternative, named Illuminus [6], uses GenCall normalized X and Y values as input. It models the data from each SNP using a four component mixture model which is fitted using an Expectation Maximization (EM) algorithm to the strength (log(*X_ij _*+ *Y_ij_*)) and contrast ((*X_ij _*- *Y_ij_*)/(*X_ij _*+ *Y_ij_*)) values to summarize the four possible states (AA, AB, BB or NC for no call). The indices *i *and *j *refer to sample and SNP respectively. Probabilities (p*_ijk_*, where *k *= 1,....,4 is the genotype index) indicating how likely a given call is correct under the model are also available. The genotype with the highest probability is the call reported to the user, and the probability provides a call confidence measure. Illuminus fits a separate three component model for X chromosome SNPs in male samples. A perturbation score is also calculated to quantify how sensitive the clustering is to changes in the initial values used in the EM-algorithm. This score serves as a SNP quality measure, and a cut-off of 0.95 and above, which equates to 95% or more of the calls agreeing after perturbation, is recommended in the Illuminus documentation. Sample quality can be measured by the percentage of calls with a posterior probability less than a threshold (0.95 is recommended). Alternatively the percentage of no calls (NC or genotype index *k *= 4) obtained for each sample can be used as a sample quality indicator. The Illuminus software is implemented in C and is available from the authors on request [6].

A third method, GenoSNP [7] is the only method which ts a within-sample model to the data. GenoSNP uses the raw (non-normalized) X and Y intensities from GenCall, which are separated by bead pool and then quantile normalized within sample. A four component mixture model similar to Illuminus is then fitted to the normalized log_2_(X*_ij _*+ 1) and log_2_(Y*_ij _*+ 1) values. SNPs from the same bead pool within a given sample are called simultaneously using the model. This approach is quite different to the other methods, which use between sample information to fit the model. In GenoSNP, a posterior probability is available for each call indicating how likely the call comes from the class assigned. This value serves as a call confidence measure. The average posterior probability across all samples for a given SNP may be used to filter SNPs, with lower average probabilities indicative of SNPs with poorer clustering under the model. A SNP cut-off of 0.95 or higher is recommended for good quality data sets, and 0.8 or higher for lower quality data sets. Likewise, the average posterior probability of all calls from a given sample can be used as a sample quality metric. A sample quality threshold of 0.9 or higher is recommended. The GenoSNP software is implemented in C and is available from the authors on request [7].

The final method in our comparison, CRLMM, was originally developed for Affymetrix data [8,9] and has recently been adapted to suit Illumina's Infinium SNP BeadChips [10]. CRLMM extracts summarized X and Y intensities directly from the idat files. For normalization, SNPs are separated based on their physical location (strip) on the BeadChip surface and simultaneously quantile normalized between channels (X and Y) and samples, using the reference distribution obtained from the HapMap training samples (Table 2). Each strip contains SNPs from multiple bead pools. After normalization, SNP-specific log-ratios (*M_ij _*= log_2 _*X_ij _*- log_2 _*Y_ij_*) and average intensities (*S_ij _*= (log_2 _*X_ij _*+ log_2 _*Y_ij_*)/2) are calculated for each array. To remove intensity dependent effects of *S *on *M*, a three-component mixture model with smoothing splines is fitted to each array via the EM-algorithm. Next, a two-level hierarchical model, with SNP-specific means and standard deviations estimated from the relevant training data set using genotype information from the HapMap project, is fitted. The intensity-dependent splines and the SNP-specific genotype means and standard deviations are combined in the model [8,9]. In general, the model assumes 3 clusters, except for X chromosome SNPs in male samples, where a 2 cluster model is used. Genotype calls are assigned by choosing the class that minimizes the negative log likelihood. CRLMM produces a number of quality assessment measures [9,13]. Per call confidence is measured using the log-likelihood ratio test from the hierarchical model. At the SNP level, the minimum distance between the heterozygote center and either of the two homozygous centers provides a SNP confidence score. A signal-to-noise ratio (SNR) for each sample assesses the separation of the three major genotype clusters within an array, with lower values indicative of poorer quality data. The CRLMM method is implemented in R [14] and is available as part of the Bioconductor project [15].

None of the methods compared make calls for the non-polymorphic copy number specific probes which are available on many Infinium chip types.

### Data sets

Each of the four algorithms was applied to the data sets described in the following sections.

#### HapMap data

We used data generated in-house at Illumina on HapMap samples [2] from 9 different chip types. We refer to these samples as *training *data sets, as they were used by two of the algorithms (GenCall and CRLMM) to train the respective models. HapMap data generated independently by a different genotyping core facility, were also analyzed. We refer to these samples as *test *data sets, as they were run independently of the training data used to calibrate two of the models. Any over-fitting of the GenCall or CRLMM models to the training data, which may give overly optimistic results for these two methods on these data sets, should not be present in the independent test samples. The number of samples for each chip-type, with a break-down by HapMap population is presented in Table 2. HapMap data has the benefit of independent calls being available [16]. These calls can be used as the gold standard for comparing the accuracy of the various calling methods.

**Table 2:** Summary of the HapMap samples analyzed by each algorithm.

Chip type	Training samples	Test samples
550 k	112 (48:12:16:36)	-
650 k	112 (48:12:16:36)	15 (0:0:0:15)
1 m	118 (49:13:17:39)	7 (3:0:0:4)
370 k Duo	115 (49:13:16:37)	45 (30:0:0:15)
1 m Duo	269 (89:45:45:90)	33 (11:3:2:17)
370 k Quad	225 (73:38:37:77)	-
610 k Quad	225 (73:38:37:77)	27 (10:5:6:6)
660 k Quad	267 (88:44:45:90)	47 (30:0:0:17)
omni1 Quad	267 (88:44:45:90)	67 (29:4:4:30)

**Total**	1,710	241

The training samples were processed in-house by Illumina. The test samples were processed by an independent core facility. The number of samples from each of the four HapMap populations represented in each collection is given in parentheses (CEU:CHB:JPT:YRI).

#### Association study data

Data from a recent genome-wide association study (GWAS) on multiple sclerosis (MS) [17] were also used in our comparison. Table 3 lists the number of samples from each batch. Different batches correspond to the various study centers in Australia and New Zealand where the samples were collected from. Each sample was analyzed using Illumina's 370 k Duo BeadChip platform processed at the same core facility within a 6 month window [18]. A total of 1,943 samples were genotyped using the four methods. We refer to this study as the MS-GWAS in the remainder of this article.

**Table 3:** The number of samples analyzed from the MS-GWAS.

Sample batch	Number of samples
1	647
2	346
3	338
4	133
5	75
6	404

**Total**	1,943

Each batch corresponds to a center where samples were recruited from.

## Results and Discussion

### Comparing accuracy using HapMap data

**Figure 1:**
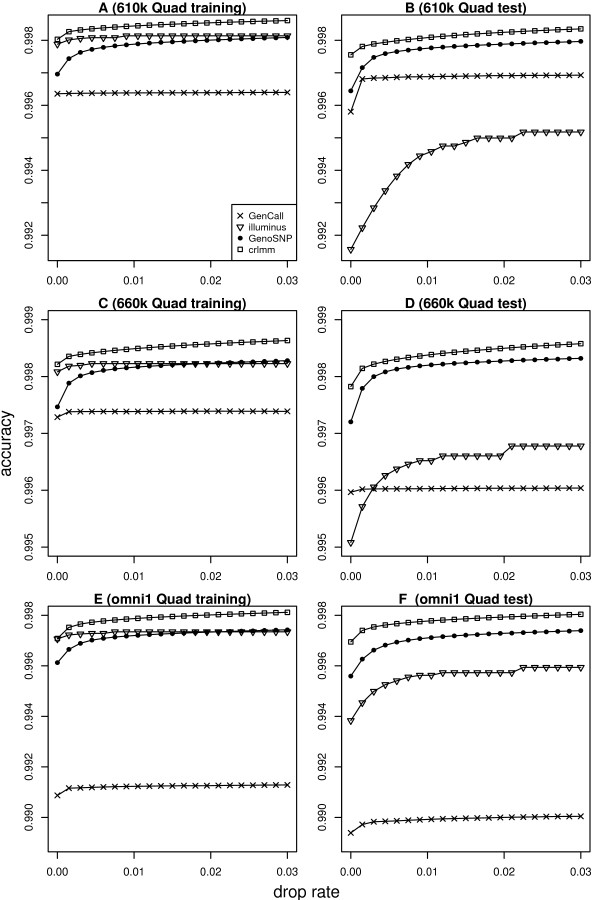
**Accuracy versus drop rate plots for the four methods tested**. Figures on the left-hand side show results for the training data sets from 610 k Quad (A), 660 k Quad (C) and omni1 Quad (E) BeadChips. Figures on the right-hand side show results for the test data sets from the 610 k Quad (B), 660 k Quad (D) and omni1 Quad (F) BeadChips. Results are shown for autosomal SNPs only. CRLMM gives slightly more correct calls than the other methods for these high density chip types. GenCall is almost always slightly worse than the other methods. GenoSNP performs very consistently between data sets, achieving accuracy slightly below CRLMM. The accuracy of Illuminus seems to improve as the number of samples available increases (accuracy starts off at around 0.992 in B with 27 samples, and increases to 0.995 in D with 47 samples and 0.998 in A and C where in excess of 200 samples are available).

For each chip type, calls from the four methods were compared with the independent genotypes available from the HapMap project. Figure 1 shows the accuracy of each algorithm for autosomal SNPs from three high density chip types. The drop rate refers to the proportion of SNPs which have been removed from the accuracy calculation based on low call confidence measures. For most chip types, CRLMM gives slightly better performance than GenoSNP and Illuminus. GenCall is generally slightly worse than the other methods. Overall differences between the four methods are small. Results for other Infinium BeadChips are broadly similar (Additional File 1: Supplemental Figure S1). We repeated these calculations on a per sample basis to obtain confidence intervals (mean +/- 2 SE) for each method. Supplemental Figure S2 in Additional File 1 shows the results for the same 3 high density chip types shown in Figure 1. In almost all instances, the confidence intervals are non-overlapping which indicates that the small differences observed between the methods are indeed significant.

**Figure 2:**
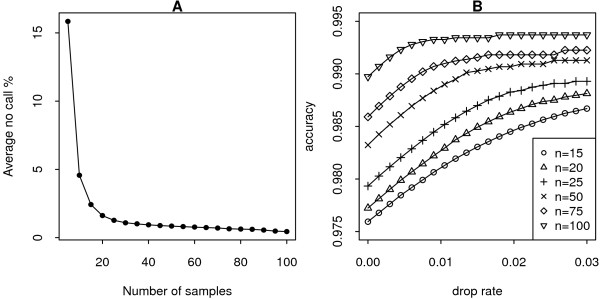
**The effect of sample size on results from Illuminus**. Illuminus average no call rate for 660 k Quad training data for varying numbers of samples (A). The average proportion of calls assigned to the 'no call' class by the model per sample declines as the number of samples included in the analysis increases. Accuracy versus drop rate from 6 different Illuminus analyses in panel A involving varying numbers of samples are also shown (B). As the number of samples analyzed increases, the accuracy measured in terms of agreement with the independent HapMap calls improves. Note that SNPs assigned to the 'no call' class are excluded from these calculations.

The performance of Illuminus is most variable among the methods, sometimes producing near the best accuracy rates, while on other data sets it is the least accurate. This phenomenon appeared to be related to sample size. To investigate this more systematically, 660 k Quad training data were analyzed using Illuminus with varying numbers of samples (from 5 to 100 in increments of 5 samples). In Figure 2A, the decrease in the number of 'no calls' as sample size increases is shown. Accuracy also improves with increasing sample number (Figure 2B). This analysis clearly demonstrates that having more samples improves the performance of Illuminus. Other methods aren't adversely affected by low numbers of samples, due to either the existence of a training step (GenCall, CRLMM) which means model parameters based on data from at least 100 samples are available, or a within-sample approach (GenoSNP) which leverages information from the many SNPs within an array to estimate the necessary parameters and make calls. Illuminus on the other hand only uses the data available, so for small data sets the model parameters will be less well estimated.

**Figure 3:**
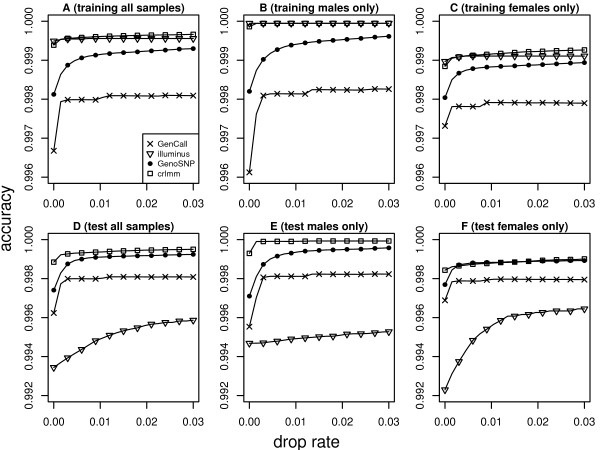
**Accuracy versus drop rate plots for the four methods tested for X chromosome SNPs**. Results are shown for all samples (A), males only (B) or females only (C) from the 610 k Quad training data and all samples (D), males only (E) or females only (F) from the 610 k Quad test data respectively. Methods with separate models for male and female samples (Illuminus and CRLMM) are generally more accurate than methods which use the same model for both sexes (GenCall, GenoSNP). Performance of Illuminus in the test data set is worse than the other three methods despite the sex-specific model. Again this is due to small sample size. In the training data set there were 121 males and 104 females, and in the test data set there were 13 males and 14 females.

We also examined which method offers the best performance on X chromosome SNPs (Figure 3), again using accuracy with the independent HapMap project calls as the gold standard. Calling algorithms which apply different models to the male and female samples (Illuminus and CRLMM) generally perform better than methods which don't (GenCall and GenoSNP). This improvement comes from higher accuracy for male samples. GenCall is generally slightly worse than the other alternatives for X chromosome SNPs. As for the autosomal SNPs, we see that the performance of Illuminus is better when large numbers of samples are available.

The accuracy versus drop rate calculations were repeated using per SNP quality measures instead of individual call confidence measures to filter entire SNPs from the analysis (Additional File 1: Supplemental Figure S3). The Illuminus perturbation score for SNP quality gives very similar accuracy to CRLMM's cluster separation metric when large numbers of samples are available (Additional File 1: Supplemental Figures S3A, S3C and S3E), while the average per SNP posterior probability of GenoSNP is slightly less accurate than these methods. For smaller sample sizes, Illuminus does less well. These measures are superior to GenCall's average GC score.

**Figure 4:**
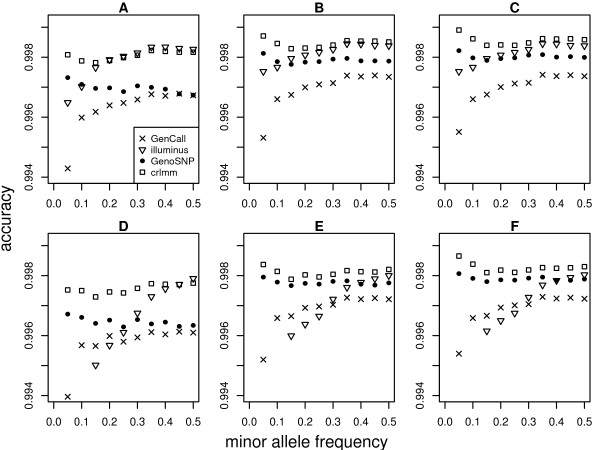
**Accuracy by minor allele frequency**. Accuracy for the 610 k Quad training data after 0% (A), 1% (B) and 2% (C) of calls with lowest confidence were removed from the analysis. The x-axis in each plot shows MAF calculated from 0.05 (5%) to 0.5 (50%) in increments of 0.05 (5%). Similar plots are shown for the 610 k Quad test data, with figures D, E and F displaying accuracy after 0%, 1% and 2% of the calls with lowest confidence were dropped from the analysis. Ignoring the overall differences in accuracy, which are consistent with the results seen in Figure 1, we see that different methods vary in performance by MAF. For example, the accuracy profile of GenCall and Illuminus increases fairly monotonically as the frequency of the rarer allele increases, with lowest accuracy obtained for SNPs with a MAF of 5% or lower. GenoSNP and CRLMM are most accurate at calling rarer alleles, and have a more consistent accuracy profile as MAF varies. These trends are consistent as more SNPs are excluded from the analysis. As we have seen in other analyses, the more samples available, the better the performance of Illuminus with higher accuracy achieved on the training data (225 samples) compared to the test data (27 samples). In figures D, E and F, the accuracies at minor allele frequencies of 5% and 10% are not plotted for Illuminus as they fall are below 0.994 (0.928 and 0.987 respectively at 0% drop rate, 0.961 and 0.992 at 1% and 0.966 and 0.993 at 2%). For Illuminus and GenoSNP, SNPs assigned to the 'no call' class are excluded from the accuracy calculations. These figures show results for autosomal SNPs only.

Stratifying accuracy by minor allele frequency (MAF) shows an interesting profile by method (Figure 4). For both the 610 k Quad training and test data sets, both GenoSNP and CRLMM have fairly consistent accuracies across the range of minor allele frequencies (from 5% - 50%). GenCall and Illuminus have poorer performance at lower minor allele frequencies, with increasing accuracy as frequency increases. These trends are consistent as the number of low confidence calls removed increases from 0% (Figure 4A and 4D) to 1% (Figure 4B and 4E) and 2% (Figure 4C and 4F). Similar trends were observed for other chip types (data not shown).

### Higher-level performance assessment

The HapMap data sets analyzed are of very high quality and not subject to the same sources of variation that affect data from genome-wide association studies. In large projects, the collection of samples and genotypes may occur over a long period of time and arrays may be processed by multiple laboratories or core facilities. We examine data from the MS-GWAS where samples have been collected from different centers and processed in batches (Table 3). GenCall, Illuminus and CRLMM were each run independently on the different batches, while GenoSNP was run one sample at-a-time. For GenCall, re-clustering was carried out by the GenTrain algorithm using the samples available instead of the default HapMap cluster information. We use these data to assess how well each method performs at flagging samples of dubious quality.

**Figure 5:**
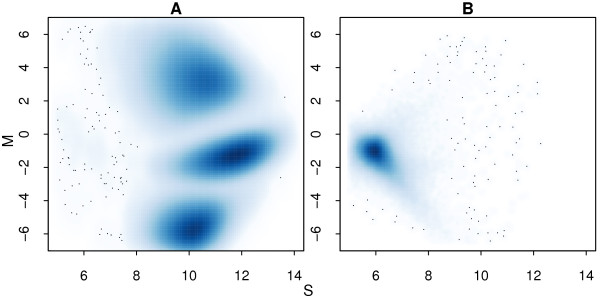
**Smoothed scatter plots of log-ratios versus average intensities for a sample run in replicate**. This figure gives an example of signal from a good quality array (A), with three well-separated clusters of points which approximately correspond to the AA (top cluster), AB (middle cluster) and BB (bottom cluster) genotypes. Signal from the same sample which is clearly of very low quality is also shown (B). In this plot we see one cluster of points, rather than the expected three. This major cluster occurs at low intensity (≈6), which is also highly unusual (intensities between 8 and 14 on the log_2_-scale are typical). In each panel, non-normalized log-ratios (*M*) are plotted on the y-axis versus average intensities (*S*) on the x-axis.

Figure 5 shows why a metric for calling poor quality samples is needed. For a typical sample, the raw signal separates into 3 major clusters (Figure 5A), whereas for a failed hybridization, distortions can be observed (Figure 5B). Having a measure which can be used to quickly flag poor quality arrays is essential in studies involving large numbers of samples. For each method, either a no call rate (GenCall, Illuminus), average posterior probability (GenoSNP) or a signal-to-noise ratio (CRLMM) can be calculated for each sample to assess quality.

**Figure 6:**
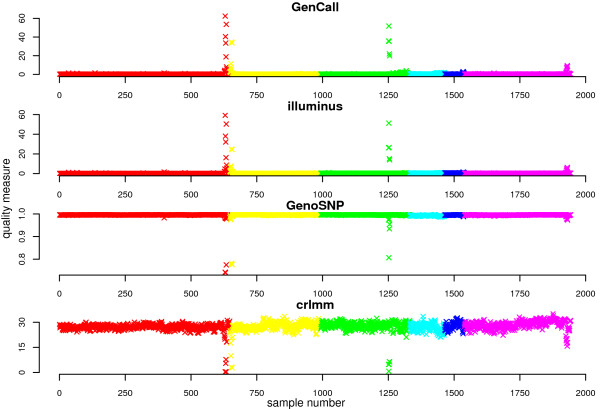
**Measures of sample quality for the MS-GWAS by genotyping method**. Samples from different batches (from 1 to 6) are plotted in different colors. For GenCall and Illuminus, the per sample no call rate (%) is used to measure sample quality. The GenoSNP per sample quality measure is the average posterior probability of all calls within a sample, with higher values (closer to 1) indicative of higher quality. In CRLMM a signal-to-noise score which measures separation between the 3 major clusters in each sample (Figure 5A) is calculated. For this measure, higher scores represent higher quality. Despite the differences in scale, all methods appear to assign the most extreme quality scores (highest values in the case of GenCall and Illuminus, and lowest for GenoSNP and CRLMM) to the same samples.

**Figure 7:**
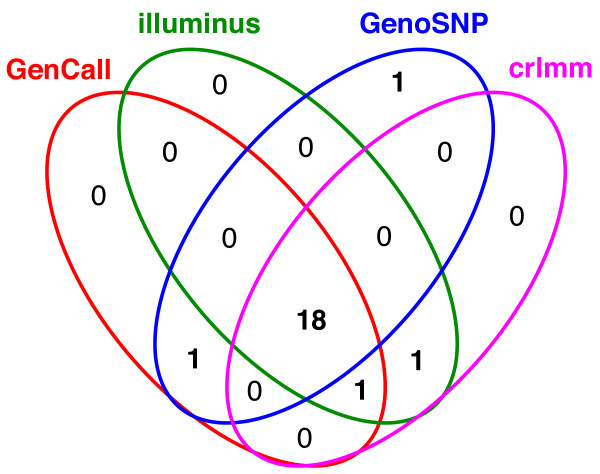
**Agreement between methods for the 20 lowest quality samples ranked by each algorithm**. All methods agree on 18 samples, GenCall, Illuminus and CRLMM all agree on a further sample. CRLMM and Illuminus or GenoSNP and GenCall both agree on another sample each. The sample flagged by GenoSNP alone was ranked just outside the worst 20 samples by the other methods (22nd, 23rd and 25th for GenCall, Illuminus and CRLMM respectively). Plots of the raw signal from 3 samples ranked amongst the worst 20 by all methods are given in Additional File 1: Supplemental Figure S5.

In Figure 6, the sample quality measures for the MS-GWAS samples are shown for each method. Despite differences in the measures used, the four methods flag many of the same samples as potential outliers. Pairs plots of the sample quality measures show this more clearly (Additional File 1: Supplemental Figure S4). As a summary, Figure 7 shows the degree of overlap between the 20 worst ranking samples obtained using the respective sample quality metrics from each method. Visual inspection of the data from many of these arrays indicates unusual signal patterns (Additional File 1: Supplemental Figure S5), which makes them good candidates to remove from further analysis.

**Figure 8:**
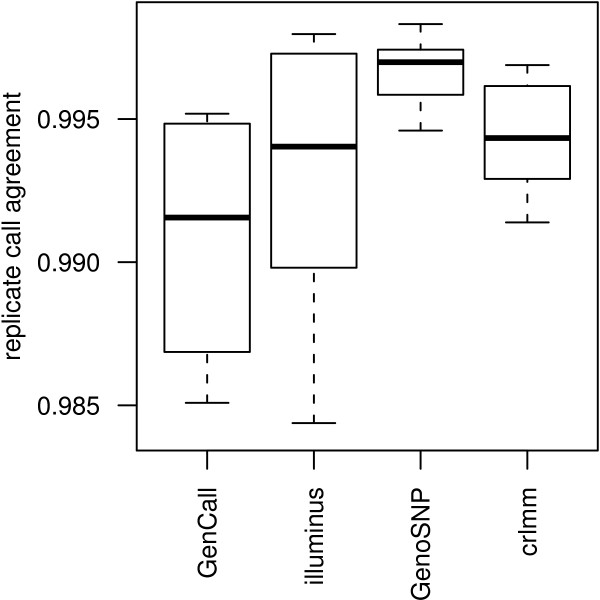
**Concordance between genotype calls from replicate samples by method**. Boxplots of the replicate concordance for 10 samples from the MS-GWAS which were analyzed using DNA derived from both saliva and blood. High concordance between replicates calls (> 98.5% agreement) is the norm. The 8th sample is an exception, due to poor quality of one of the replicates (Figure 5). For this pair of samples, the concordance values are 16.6%, 15.6% and 40.8% for GenCall, Illuminus and CRLMM respectively (values not plotted as they are off the scale). GenoSNP did not produce calls for one of the samples (Figure 5B), so concordance could not be calculated for this replicate pair.

The agreement between calls made on replicate samples was also assessed for each method. In Figure 8, the agreement of calls between 10 replicate samples analyzed from DNA extracted from blood and saliva from the MS-GWAS are shown. High levels of agreement (> 98.5% of calls) were obtained for all methods. The concordance from one pair of samples (Figure 5) is not shown, due to the poor quality of one of the replicate samples (Figure 5B - sample quality measures of 56.8%, 19.8% and 0.34 for GenCall, Illuminus and CRLMM respectively were obtained for this sample, which are extreme values on the respective scales shown in Figure 6). GenoSNP does not produce calls for this sample. Presumably, the lack of separation between the three clusters causes numerical problems for the model.

The computing resources used to run each method on a set of samples from the MS-GWAS were also examined. Table 4 shows the time taken and memory usage of each algorithm. We note that GenCall was only available under Windows, and was run on a different computer to the other methods, which means our results cannot be directly compared. In spite of this, we can say that CRLMM is the fastest method of the three which were run on the same linux system, followed closely by Illuminus and then GenoSNP. CRLMM is by far the biggest consumer of RAM using approximately three times as much memory as Illuminus. In contrast, GenCall and GenoSNP use very little RAM.

**Table 4:** Summary of the computing resources required by each method.

Software	Time taken (mins)	Peak memory usage (GB)
GenomeStudio (v 1.1.0)*	230	0.75
GenoSNP^†^	370⋄	0.09
Illuminus^†^	38⋄	12.2
CRLMM (v 1.2.4)^†^	28	38.2

These figures are based on the analysis of a set of 346 samples (batch 2) from the MS-GWAS.

* run on a PC (Pentium quad core, 3 Ghz computer with 2 GB RAM) with file-based storage enabled

^† ^run on a quad-core AMD Opteron 2.7 Ghz CPU linux machine with 64 GB RAM

^⋄ ^timing does not include time taken by GenomeStudio to output X and Y data used as input for these methods (30 mins and 0.65 GB RAM on the PC we used)

## Conclusions

Our study represents the largest comparison of genotyping methods for Illumina's Infinium BeadChip platform to date. We examined the performance on data sets varying in size from tens to nearly 2000 samples from a wide range of chip types.

Despite the differences in approach, the four methods compared generally o er similar performance in terms of accuracy with high quality HapMap data (> 99% agreement), when call or SNP-specific quality scores were used to filter data. CRLMM is marginally better than GenoSNP and Illuminus (when sample size is large enough), followed by GenCall. Each method also gives high concordance between replicate samples (> 99% on average). Variations in the ability of different methods to correctly recover calls from SNPs with low minor allele frequency were observed, with CRLMM and GenoSNP outperforming GenCall and Illuminus for SNPs with the lowest MAF. This points to the benefit of borrowing information between SNPs. In GenoSNP, this is done explicitly by using the many observations from a given bead pool to estimate parameters in the mixture model and assign genotypes. For CRLMM, there will be little information from the training data set on the heterozygous and homozygous cluster locations involving the minor allele. However, since the SNP-specific parameters are updated by an empirical Bayes shrinkage procedure, more weight will be placed on the priors in these situations. These priors are derived from other SNPs in the data set. Both approaches cope better than methods which model the data from each SNP independently (GenCall and Illuminus) when MAF is low. This issue will be important as arrays include more rare variants (MAF < 5%), such as SNPs discovered in the 1000 Genomes Project [19].

We observed that the performance of Illuminus depends upon the number of samples available for the analysis, with larger sample sizes (≥50), giving better results in terms of no call rate and accuracy. For genome-wide association studies, low sample numbers are not likely to be a problem, however for linkage studies, which are often much smaller (< 10 samples), Illuminus would not be the method of choice, unless the samples can be analyzed within a larger batch of the same chip type. All other methods can handle data from small-scale projects without compromising performance.

We note that relative to the time expended recruiting and collecting samples and processing arrays, the time taken to run each algorithm is insignificant, with slightly longer processing times unlikely to be a major factor effecting the choice of method. The ability to parallelize genotyping between multiple processors is a simple way to reduce the time taken to process samples. All four algorithms allow parallelization. By default, GenomeStudio divides the analysis between the available processors, splitting on sample or SNP depending upon the stage of the analysis. For GenoSNP, which processes samples one-at-a-time, parallelization is trivial; the user can easily divide the samples between the processors available. For Illuminus and CRLMM, the between-sample nature of the modelling, means that parallelization requires SNPs to be split between processors. This feature is available as an option in both algorithms. In CRLMM, the parallelization is handled using the *snow *package in R.

As for timing, researchers involved in large scale studies are likely to have access to high performance computing facilities, which means that large memory requirements of methods like CRLMM, and to a lesser extent Illuminus are not likely to pose a limitation. In the most recent version of CRLMM, the memory footprint can be reduced through use of the *ff *package in R. This package utilizes available disk space instead of RAM when RAM is limited to store the raw data and genotyping output.

One drawback of the current implementation of CRLMM is its reliance on training data to calibrate the model parameters, which means that for customized genotyping, or genotyping in non-model organisms (such as cow, pig and chicken), it cannot be applied due to a lack of availability of HapMap-like training data. We are currently investigating modifications to CRLMM to ensure it can be applied in such settings. While GenCall also includes a training step on HapMap data for the chip types analyzed in this paper, it can also work in an unsupervised manner, where it estimates cluster centers using the data available without the need for any prior information. Illuminus and GenoSNP can also be used on BeadChips containing customized human SNP sets or SNPs from other diploid organisms.

Further work would be to extend the comparison to include newer genotyping methods, such as BeagleCall [12], which adds an extra layer of haplotype information to the genotype calling process. The improvements offered by the recently released update to the GenTrain clustering algorithm (version 2) are also of interest. GenTrain2 was not used in this study, as output from this software was unavailable for any of the data sets analyzed. Since most studies published to date will be based on the older version of GenCall, our comparison is still relevant.

## Authors' contributions

RL and MER performed the analysis. MER wrote the manuscript. BSC was involved in software implementation and improvement and provided feedback on the manuscript. The ANZgene Consortium provided data used in this study. RAI oversaw the study and finalized the manuscript. All authors' read and approved the final manuscript.

## Supplementary Material

Additional file 1 **Supplemental Figures**. Click here for file
