# Supplementary material for: Comparing genotyping algorithms for Illumina's Infinium whole-genome SNP BeadChips
Source: BMC Bioinformatics. 2011 Mar 8;12:68. doi: 10.1186/1471-2105-12-68 (PMC3063825; doi:10.1186/1471-2105-12-68)
Supplement: Additional file 1 — Supplemental Figures. [file 1471-2105-12-68-S1.PDF]

Additional File 1 – Supplemental Figures

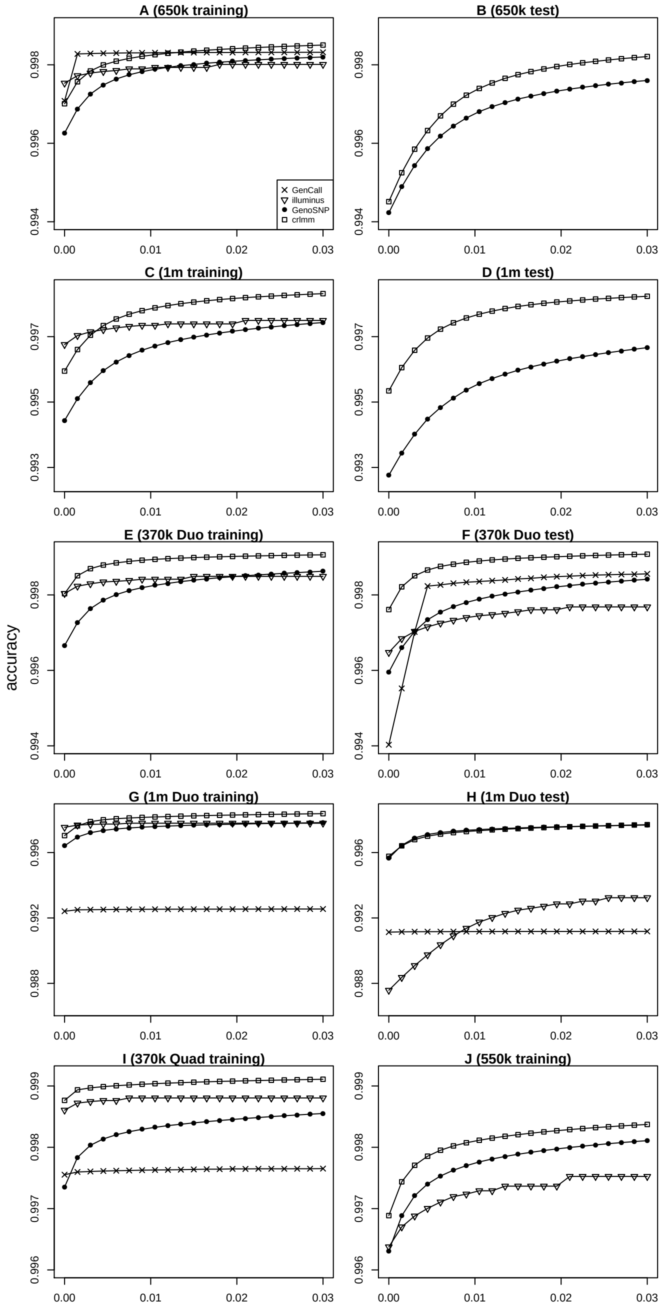

Supplemental Figure S1: Accuracy versus drop rate for the four methods tested for the remaining chip types. Plots for the 650k training (A), 650k test (B), 1m training (C), 1m test (D), 370k Duo training (E), 370k Duo test (F), 1m Duo training (G), 1m Duo test (H), 370k Quad training (I) and 550k training (J) data sets are given. SNPs were dropped on the basis of low call confidence values (as per Figure 1 in the main article). For some data sets (B, C, D, E, J), GC scores were unavailable, so results for GenCall could not be plotted. For data sets with 15 or fewer samples (B, D), Illuminus results are not plotted as the accuracy was lower than the minimum values displayed on these plots.

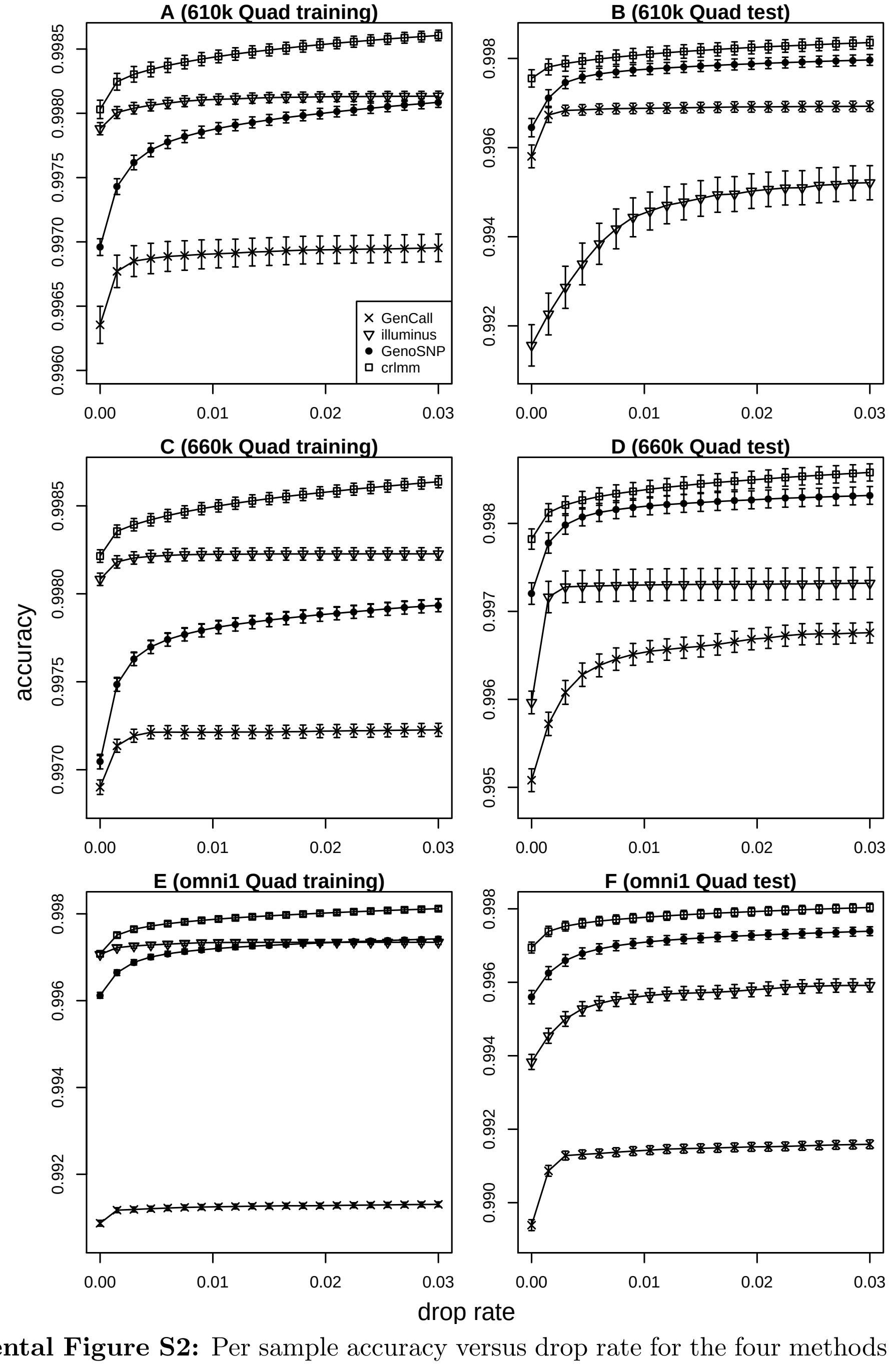

Supplemental Figure S2: Per sample accuracy versus drop rate for the four methods tested on 3 high density chip types. Figures on the left-hand side show results for the training data sets from 610k Quad (A), 660k Quad (C) and omni1 Quad (E) BeadChips. Figures on the right-hand side show results for the test data sets from the 610k Quad (B), 660k Quad (D) and omni1 Quad (F) BeadChips. The per sample analysis allows confidence intervals for accuracy (mean +/− 2 SE) to be calculated for each method at various drop rates. Results are shown for autosomal SNPs only.

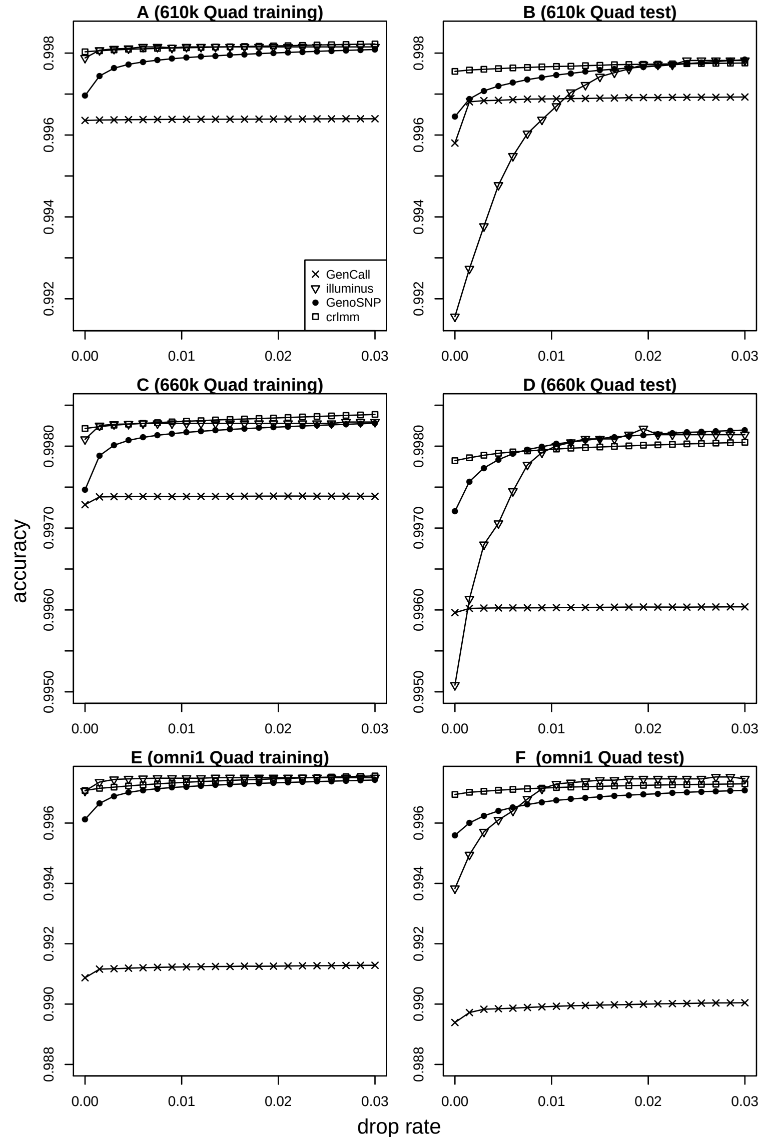

Supplemental Figure S3: Accuracy versus drop rate for the four methods tested. In these plots, SNPs are ranked using SNP confidence measures, rather than call confidence. Results for the 610k Quad training (A), 610k Quad test (B), 660k Quad training (C), 660k Quad test (D), omni1 Quad training (E) and omni1 Quad test (F) data sets are shown.

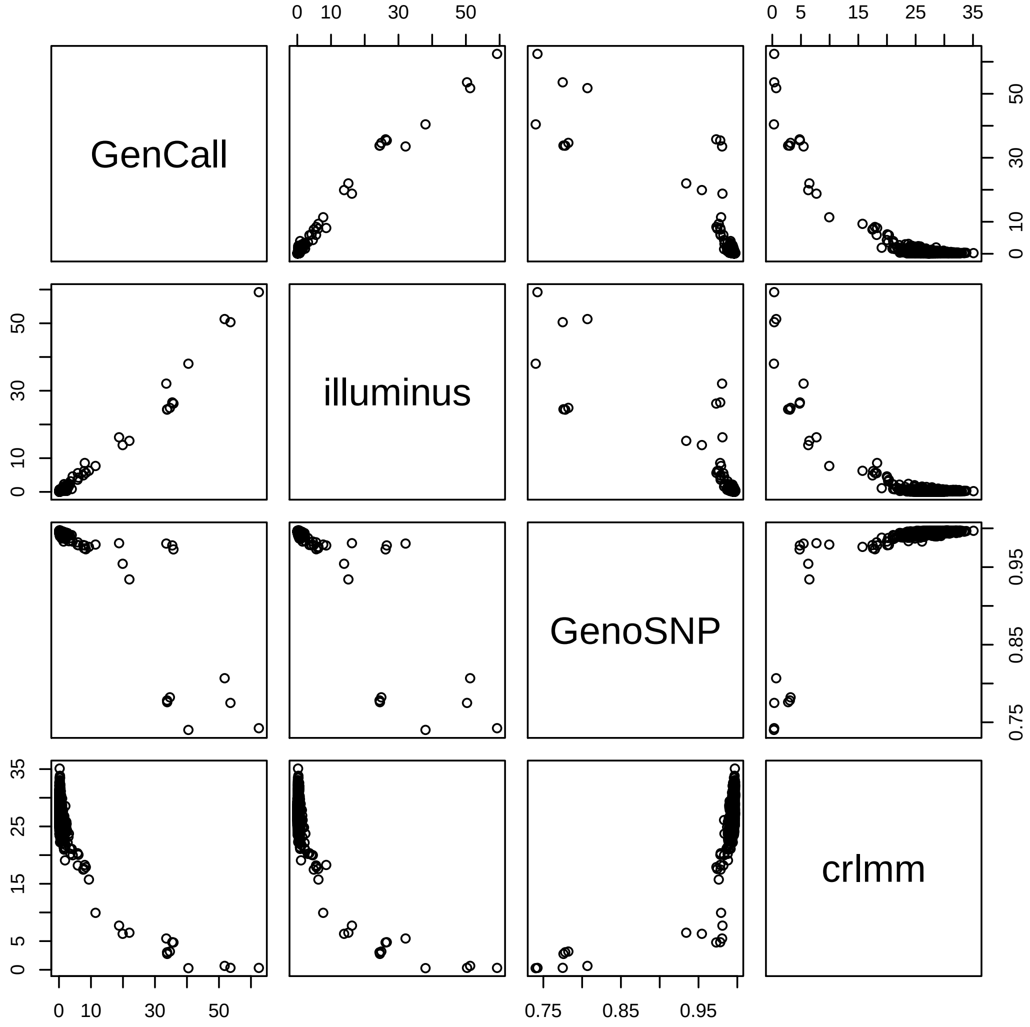

Supplemental Figure S4: Pairs plot of the sample quality measures from each algorithm for the 1,943 samples from the MS-GWAS. This figure shows the high level of agreement between outlier samples (i.e. samples with extreme values on the respective scales, either high no call rates for GenCall or Illuminus, low average posterior probability for GenoSNP or low signal-to-noise ratio for CRLMM) identified by each method. Figure 7 in the main article shows the degree of overlap between the four methods when we restrict our analysis to the 20 samples with the most extreme (worst) sample quality measures.

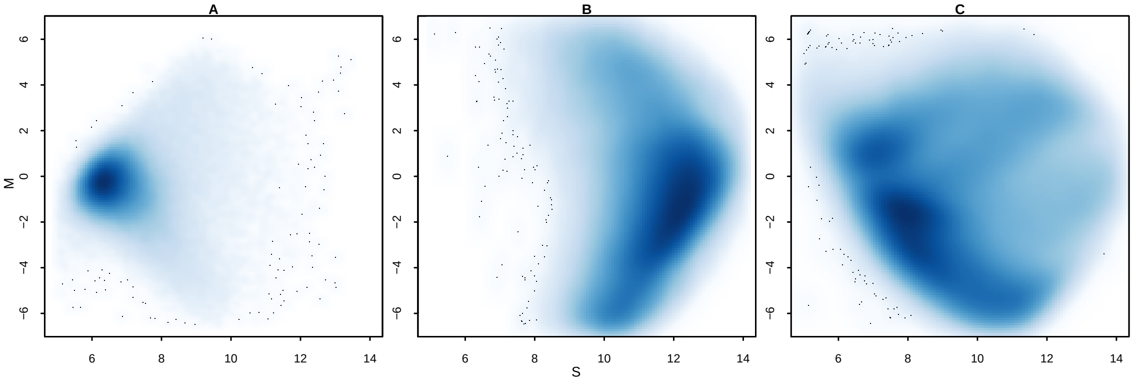

Supplemental Figure S5: Smoothed scatter plots of non-normalized log-ratios (M) versus average intensities (S) for three samples ranked amongst the worst 20 samples in terms of quality by all methods. In each case, uncharacteristic signal patterns can be observed, with either too few clusters (A) or smears which show poor separation between the three major clusters (B and C). In a good quality sample, these clusters are well separated (see Figure 5A in the main article).
